# Integrin α_IIb_β_3_ Transmembrane Domain Separation Mediates Bi-Directional Signaling across the Plasma Membrane

**DOI:** 10.1371/journal.pone.0116208

**Published:** 2015-01-24

**Authors:** Ping Hu, Bing-Hao Luo

**Affiliations:** Department of Biological Sciences, Louisiana State University, Baton Rouge, Louisiana, United States of America; University of California Berkeley, UNITED STATES

## Abstract

Integrins play an essential role in hemostasis, thrombosis, and cell migration, and they transmit bidirectional signals. Transmembrane/cytoplasmic domains are hypothesized to associate in the resting integrins; whereas, ligand binding and intracellular activating signals induce transmembrane domain separation. However, how this conformational change affects integrin outside-in signaling and whether the α subunit cytoplasmic domain is important for this signaling remain elusive. Using Chinese Hamster Ovary (CHO) cells that stably expressed different integrin α_IIb_β_3_ constructs, we discovered that an α_IIb_ cytoplasmic domain truncation led to integrin activation but not defective outside-in signaling. In contrast, preventing transmembrane domain separation abolished both inside-out and outside-in signaling regardless of removing the α_IIb_ cytoplasmic tail. Truncation of the α_IIb_ cytoplasmic tail did not obviously affect adhesion-induced outside-in signaling. Our research revealed that transmembrane domain separation is a downstream conformational change after the cytoplasmic domain dissociation in inside-out activation and indispensable for ligand-induced outside-in signaling. The result implicates that the β TM helix rearrangement after dissociation is essential for integrin transmembrane signaling. Furthermore, we discovered that the PI3K/Akt pathway is not essential for cell spreading but spreading-induced Erk1/2 activation is PI3K dependent implicating requirement of the kinase for cell survival in outside-in signaling.

## Introduction

Integrins are single transmembrane (TM) α-β heterodimeric cell adhesion receptors with each subunit comprised of a large extracellular domain, a single TM helix, and a short cytoplasmic domain [1–4]; integrins can transmit bidirectional signals across the plasma membrane. Studies have shown that in the resting state, the ectodomains adopt a bent conformation that is stabilized by specific α/β interfaces that exist in the extracellular, TM, and cytoplasmic domains. Integrins can be activated through an “inside-out” signaling pathway that results in an extended conformation with high affinity for ligands [5]. Upon interacting with multivalent extracellular ligands, integrins can transmit signals inward, i.e. outside-in signaling, that influence biological processes such as cell mobility, proliferation, and differentiation [6].

The integrin TM/cytoplasmic domains regulate integrin affinity and mediate downstream signal transduction. Association of the TM/cytoplasmic domains between the α and β subunits is critical for maintaining integrins in the low-affinity state. Intracellular signals that impinge on the cytoplasmic domain destabilize αβ association and result in integrin activation [7–15]. Recent research has revealed the structures of both the associated and isolated monomers of the TM/cytoplasmic domains and greatly advanced our understanding of TM activation [16–22]. In the resting state, ridge-in-groove packing of the TM domain and the GFFKR motif in the α subunit cytoplasmic domain are important for αβ association, whereas binding of intracellular molecules such as talin [23] dissociates the αβ TM/cytoplasmic domains and leads to integrin activation.

TM separation has also been reported to be required for outside-in signaling [15,24]. Previous studies indicated that clasping of the TM domain abolished cell spreading and focal adhesion (FA) formation [24]. However, the research left a critical question unanswered: if TM domain separation is essential or it is cytoplasmic domain dissociation that actually matters since TM clasping can probably cause defects in cytoplasmic domain dissociation. TM separation is likely an intermediate conformational change that either couples cytosol activation with ectodomain extension/opening in integrin activation or mediates cytoplasmic domain separation upon immobilized ligands binding in outside-in signaling. Therefore, TM separation might not be truly “essential” in outside-in signaling and can be bypassed by artificially dissociating cytoplasmic domains. To reach a comprehensive understanding of integrin transmembrane signaling, especially outside-in signaling, it is extremely important to dissect the role of TM separation from cytoplasmic domain separation in integrin signaling. Besides the β subunit, the α subunit has also been reported to be important for outside-in signaling, especially for paxillin signaling [25,26]. It has been shown that binding of paxillin to the α_4_ and α_9_ integrin cytoplasmic tails negatively affects cell spreading but can promote cell migration [25,27]. However, since paxillin also binds to β_3_ integrin *in vitro* [28] and no direct interactions between paxillin and β_3_ integrin partners (α_v_ and α_IIb_) have been reported, we speculated that the α cytoplasmic tail might be dispensable for outside-in signaling mediated by the β_3_ integrin families. Kinase activation has been regarded as an essential step in integrin outside-in signaling; it is involved with a complex network and affects not only mechano-related cellular events such as spreading and migration but also cell survival and proliferation [29]. Many kinases have been reported to be important for outside-in signaling including focal adhesion kinase (FAK) [30], Src family kinase [31], and the PI3K/Akt pathway [32,33]. However, studies using FAK inhibitor or an unactivatable Src kinase mutant achieved nearly normal cell spreading, a signature of normal outside-in signaling [34,35], suggesting that spreading does not require high enzymatic activity of the kinases. This conflicting evidence makes it difficult to understand how the ligand binding induced integrin TM/cytoplasmic domain conformational changeaffects the signaling cascade.

In this study, we established CHO cell lines that stably express wild type and mutant α_IIb_β_3_ to study integrin signaling. CHO cells which have been widely used to study integrin signaling [15,24,31,36,37]. Although this cell line does not express surface receptors that response to extracellular stimuli which activate integrin through inside-out signaling pathway, in our study, the upstream inside-out signaling pathway was bypassed by α_IIb_ subunit truncation and thus lacking of platelet surface receptors unlikely affect our study in integrin bidirectional signaling. Lipid composition has been implicated to be important in integrin activation and clustering [38]. However, there is no evidence suggesting that the lipid composition differences between platelets and CHO cells can alter the integrin signaling. Studies showed that CHO cells that expressed integrin α_IIb_β_3_ could transmit integrin signals [24,31,36,37], making the cell line a suitable model for studying α_IIb_β_3_ signaling.

Our results showed that TM domain dissociation is a downstream conformational change following cytoplasmic dissociation in inside-out activation and a prerequisite of ligand-induced outside-in signaling. TM-clasping severely impaired outside-in signaling, leading to defects in cell spreading, migration, FA formation, and FAK activation. These defects could not be rescued by a whole α_IIb_ cytoplasmic domain truncation. Our study also indicated that α_IIb_ does not play an important role in outside-in signaling. Removal of the entire α_IIb_ cytoplasmic tail did not alter recruitment and activation of FAK and paxillin as well as actin polymerization.

## Materials and Methods

### Cell lines, Plasmid Construction, Expression, and Immunoprecipitation

CHO-K1 cells were commercially purchased from ATCC (ATCC-CCL-61, Manassas, VA). Cells were maintained in MEM-α medium (Life Technologies, Grand Island, NY) supplemented with 10% FBS, 1% None-Essential Amino Acids, 1% L-Glutamine, 1% Sodium Pyruvate and 1% Penicillin-Streptomycin (All from Life Technologies, Grand Island, NY). Plasmids containing wild type and truncated human integrin α_IIb_ were subcloned into pEF/V5-HisA; β_3_ was subcloned into pcDNA3.1/Myc-His (+) [36]. Mutants were created by QuikChange kit (Stratagene, La Jolla, CA). Constructs were stably transfected into CHO-K1 cells using FuGENE transfection kit ((Roche Diagnostics, Indianapolis, IN). The expression levels of α_IIb_β_3_ were determined by flow cytometry staining with the following monoclonal antibodies (mAbs): AP3 (anti-β_3_ mAb, American Type Culture Collection), 7E3 (anti-β_3_ mAb), and 10E5 (anti-α_IIb_ mAb, gifted by B. S. Coller, Rockefeller University, NY). To characterize disulfide bond formation, cells were labeled with S^35^ and lysed as described [11]. Lysates were immunoprecipitated with mAb 10E5 and protein G-Sepharose and subjected to SDS-PAGE and radioautography.

### Soluble Ligand Binding Assay

Soluble binding of the integrin ligand-mimetic IgM PAC-1 (BD Biosciences, San Jose, CA) and Alexa Fluor 488-labeled human fibrinogen (Enzyme Research Laboratories, South Bend, IN) was determined as previously described [39]. Cells suspended in 20 mM HEPES-buffered saline (pH 7.4) (HBS) supplemented with 5.5 mM glucose and 1% BSA were incubated on ice for 30 min with 10μg/mL PAC-1 or 20μg/mL labeled fibrinogen in the presence of either 5mM EDTA, 5mM Ca^2+^, or 1mM Mn^2+^. Cells were also stained in parallel with 10E5. Binding activity is presented as the percentage of the mean fluorescence intensity (MFI) of PAC-1 or fibrinogen staining relative to the MFI of 10E5 staining.

### Cell spreading and Microscopy

Glass-bottom six-well plates (MatTek Corporation, Ashland, MA) were coated with 20 μg/mL fibrinogen in phosphate-buffered saline (PBS) at pH 7.4 overnight at 4°C and blocked with 1% BSA at room temperature for 1 h. The cells were detached with trypsin/EDTA and washed with serum-free DMEM containing 0.5mg/mL soybean trypsin inhibitor (EMD Millipore Corporation, Billerica, MA). Cells in suspension were incubated on ice for 30 minutes in DMEM containing 10 μg/mL mouse mAb LM609 before seeding on fibrinogen coated plates. The cells were seeded with or without 1 mM DTT and incubated at 37°C for 1 h. Cells were then washed three times with PBS, fixed with formaldehyde, and subjected to differential interference contrast (DIC) imaging. For confocal microscopy, cells were seeded in DMEM medium containing either blank DMSO, 1.5 μM/mL Wortmannin (Cell Signaling Technology, Danvers, MA), or 15 μM/mL PP1 (EMD Millipore, Billerica, MA) for 1 h. After fixation, cells were permeabilized with 0.1% Triton X-100 and blocked with 1% BSA for 30 min. Phosphorylated FAK or paxillin were identified using rabbit polyclonal FAKpY397 antibody (Millipore, Billerica, MA) or rabbit paxillinpY31 polyclonal antibody (Life Technologies, Grand Island, NY), respectively. FA formation and actin filament organization were detected with Actin Cytoskeleton/Focal Adhesion Staining Kit (Millipore, Billerica, MA). Dylight 488 or dylight 633 conjugated secondary antibodies (Thermo Scientific Pierce, Rockford, IL) were used to stain primary antibodies.

Both DIC and confocal microscopy were conducted using a Leica TCS-SP2 spectral confocal system with a 63×/1.4 NA oil objective. Average cell areas were calculated from more than 100 randomly picked adherent cells and measured in pixels by ImageJ.

### Immunoblotting

Cells were seeded onto 10cm petri dishes coated with 20 μg/mL fibrinogen or 1% BSA. After 1 h spreading at 37°C, adherent cells were washed three times with ice-cold PBS and lysed with TBS with 1% NP-40, 0.25% sodium deoxycholate, 1mM EDTA, 1mM Na3VO4, and 1mM NaF supplemented with protease, tyrosine phosphatase and serine/threonine phosphatase inhibitor cocktails (Sigma Aldrich, St. Louis, MO)]. Lysates were subjected to reducing SDS-PAGE and transferred to PVDF membrane. The total amount of the kinases was determined by mouse monoclonal anti-FAK (BD Bioscience, San Jose, CA), rabbit monoclonal anti-Akt, or anti-Erk1/2 (Cell Signaling Technology Inc., Danvers, MA) antibody, respectively, and activation of the kinases was tested by rabbit monoclonal anti-FAKp397, anti-Aktp473, and anti-Erk1/2p202/204 respectively (Cell Signaling Technology Inc., Danvers, MA). Mouse monoclonal GAPDH antibody (Sigma-Aldrich, St. Louis, MO) was used to determine total protein concentration.

### Wound-healing assay

Six-well plates were pre-coated with 20 μg/mL fibrinogen followed by blocking with 1% BSA. Stable transfectants were then seeded in the wells, cultured to a confluent monolayer and serum starved overnight at 37°C. Artificial wounds were carefully created with sterilized micropipette tips. Cells were then washed twice with serum-free MEM-α medium and cultured in complete medium at 37°C. Closure of the artificial wounds was observed and recorded every 4 h for 12 h using the 10 × lens of an Olympus IX81 microscope. The wound closure rates were normalized to the original wound areas.

## Results

### Expression of wild-type and mutated α_IIb_β_3_ in CHO cells

**Figure 1:**
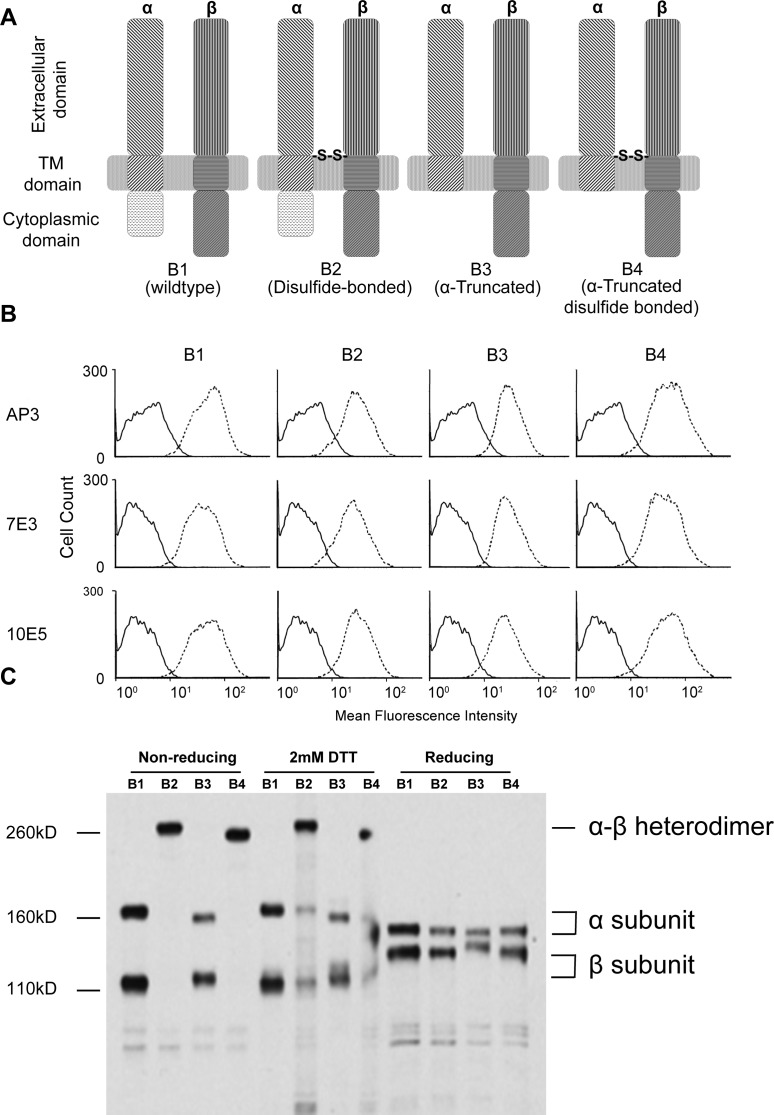
All α_IIb_β_3_ mutants were successfully expressed with high disulfide-bridge formation efficiency. (A)Integrin α_IIb_β_3_ constructs used in our study. B1: Wild type integrin α_IIb_β_3_; B2: Disulfide-bonded α_IIb_β_3_(α_IIb__W968C, β_3__I693C); B3: α truncated α_IIb_β_3_(α_IIb_1-990); B4: α truncated, disulfide-bonded α_IIb_β_3_ (α_IIb_1-990_W968C, β_3__I693C). (B) Cell lines stably expressing WT and mutated integrin α_IIb_β_3_ as evaluated by three different monoclonal antibodies. B1 (WT α_IIb_β_3_), B2 (disulfide-bonded α_IIb_β_3_ mutant), B3 (α-truncated α_IIb_β_3_), and B4 (disulfide-bonded α-truncated α_IIb_β_3_). The solid line and dashed line represent untransfected CHO-K1 cells and stable transfectants, respectively. AP3, 7E3, and 10E5 are mAbs targeting different domains of the α_IIb_β_3_ heterodimer. (C) Disulfide bonds formed between α and β subunits with high efficiency. Cells labeled with S^35^ were lysed and subjected to immunoprecipitation by anti-α_IIb_ mAb 10E5. Immunoprecipitated protein was then resolved by SDS-PAGE and visualized by radioautography. 2mM DTT was used to reduce the disulfide bridge in TM-clasped mutants.

A previous study has shown that the double cysteine mutant α_IIb__W968C and β_3__I693C reversed inside-out activation and kept integrin in the low affinity state [11]. It has also been shown that the disulfide-bonded mutant abolished integrin outside-in signaling [24]. However, it is possible that the disulfide bridge constrained the cytoplasmic domains from separation which might be a key factor in integrin bidirectional signaling. To dissect the role of the TM and cytoplasmic domains and further study the details of integrin bi-directional signaling, we introduced mutants that removed the α_IIb_ cytoplasmic domain with or without the TM disulfide clasp. We established CHO cells stably transfected with each of the four different α_IIb_β_3_ constructs (Fig. 1A): WT (B1), disulfide-bonded mutant (α_IIb__W968C, β_3__I693C; B2), truncated mutant (α_IIb_1-990; B3), and disulfide-bonded truncated mutant (α_IIb_1-990_W968C, β_3__I693C; B4). Four cell lines were generated after single-cell sorting and the integrin α_IIb_β_3_ expression levels were determined by staining with three specific mAbs: anti-β_3_ mAbs AP3 and 7E3, and an anti-α_IIb_ mAb 10E5 (Fig. 1B). Flow cytometry showed that the integrins from all three mutants were highly expressed and correctly folded, and the expression levels were comparable to each other. Untransfected CHO cells did not express endogenous α_IIb_β_3_. Immunoprecipitation of [^35^S] labeled proteins with the α_IIb_-specific mAb 10E5 (Fig. 1C) confirmed that the efficiency of disulfide formation in the disulfide-bonded mutants (B2 and B4) was about 100%. These results indicated that clasping and truncating integrin α_IIb_β_3_ did not affect overall protein folding; the high efficiency of disulfide formation made the cell lines ideal for studying the role of TM dissociation in integrin signaling.

### TM domain separation is essential for high affinity α_IIb_β_3_

**Figure 2:**
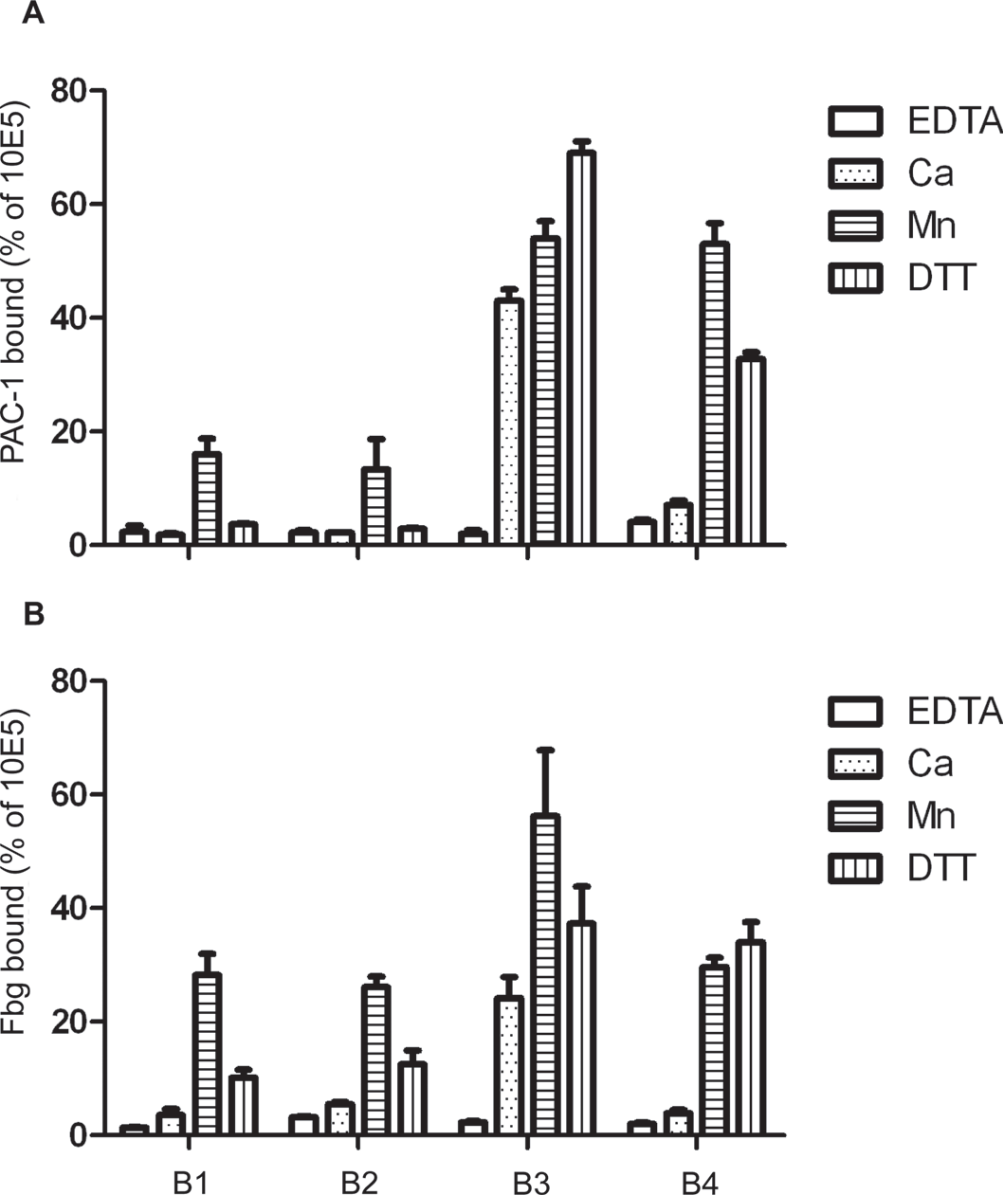
TM domain disassociation is required for cytoplasmic domain dissociation-induced high affinity integrin as measured by soluble ligand binding. Binding of ligand mimetic mAb PAC-1(A) and fibrinogen (Fbg, B) to B1 through B4 cells in the presence of either EDTA (5mM), Ca^2+^ (5mM), Mn^2+^ (1mM), or Ca^2+^(5mM) with DTT (4mM) as indicated. Error bars represent standard deviation (S.D.) from three independent assays.

We determined the ability of the integrin α_IIb_β_3_ mutants to bind ligands including PAC-1, a ligand mimetic mAb, and fibrinogen (Fig. 2A and B). In the presence of Ca^2+^, both WT (B1) and the disulfide-bonded mutant (B2) showed similar low ligand binding, whereas the α-truncated mutant (B3) exhibited much higher binding. However, the TM disulfide-bridge clasp of the mutant B4 reduced ligand binding to basal level. Reduction of the disulfide bond with DTT drastically increased ligand binding of the mutant. These results indicated that TM domain separation is required for cytoplasmic domain dissociation induced high affinity integrin.

### TM domain separation is indispensable for α_IIb_β_3-_mediated outside-in signaling

**Figure 3:**
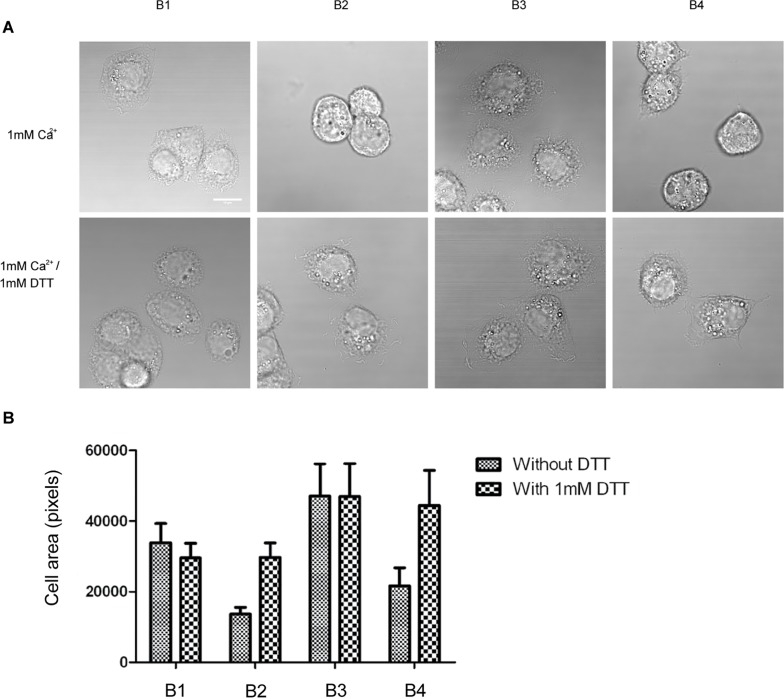
TM domain separation is required for cell spreading. (A) Stably transfected cells were seeded on immobilized Fbg (20μg/mL) with or without 1mM DTT for 30min at 37°C and visualized using differential interference contrast (DIC). Images are representatives from one of three independent assays. White bar: 10μm. (B) Average areas of adherent cells were quantified by pixel. The error bars represent S.D. from 100 randomly chosen cells.

**Figure 4:**
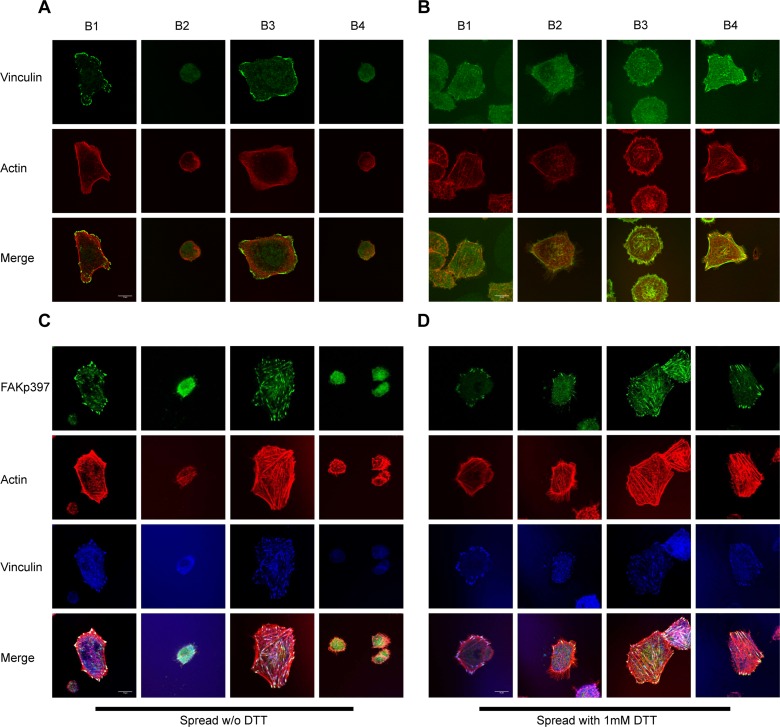
TM domain separation is required for FA formation, actin fiber organization, FAK activation and recruitment to FA sites in outside-in signaling. (A) Clasping of TM domains ablated FA formation and disrupted actin filaments organization. Note that α truncation led to an even distribution of FAs around adherent cells. (B) Treatment with 1mM DTT largely restored cell spreading and FA assembly. Green: focal adhesions (labeled with anti-vinculin antibody); Red: actin filaments (labeled with TRITC-conjugated Phalloidin). White bar: 10μm. (C) Activated FAK (FAKpY397) was recruited normally to FAs in WT (B1) and the α-truncated mutant (B3), but recruitment was abolished by clasping the TM domains (B2 and B4). (D) Treatment with 1mM DTT restored recruitment of phosphorylated FAK to FAs. Green: FAKpY397; Red: actin filaments; Blue: FA marker vinculin. White bar: 10μm.

The binding of integrin to immobilized multivalent ligands can induce integrin clustering and outside-in signaling. To dissect the role of the TM and cytoplasmic domains in outside-in signaling, we used these integrin mutants to perform a cell spreading assay on immobilized fibrinogen (Fig. 3). Clasping the TM domain abolished cell spreading (Fig. 3, B2 and B4) regardless of the presence of the α subunit cytoplasmic domain. However, when the disulfide bond was reduced, cells spread similarly to the wild type cells (B1). The α-truncated mutant (B3) showed enhanced cell spreading compared to the WT, which may be caused by the higher ligand binding affinity of the mutant or by disruption of the α subunit-paxillin interaction, previously reported to negatively regulate cell spreading in other integrins [40]. Confocal microscopy (Fig. 4A and B) showed that both disulfide bonded mutants (B2 and B4) exhibited defective FA formation and actin filament organization. These defects were restored by DTT treatment, suggesting that cytoplasmic domain separation alone is insufficient for transmitting the outside-in signal. We further studied activation and recruitment of FAK to FAs (Fig. 4C and D). We observed prominent recruitment of FAKpY397 to FAs (Fig. 4C) in WT (B1) and the α truncated mutant (B3) but not in the TM-clasped mutants (B2 and B4). DTT treatment partially restored the recruitment (Fig. 4D), suggesting that the TM domain separation is essential for outside-in signaling initiation and transduction.

In the study, we also noticed that the B3 mutant showed more and larger focal adhesions (S1 Fig.) and the FAs were distributed evenly at the edge of the cells (data not shown), suggesting loss of polarity during cell spreading. This result also implied that the mutant might be defective in directional cell migration; we observed defective cell migration in an *in vitro* wound healing assay (S2 Fig.). This phenomenon may be caused by lack of the α cytoplasmic tail, which was reported to inhibit cell spreading and promote cell migration in lymphocytes, or by high affinity ligand binding of the mutant [25,40].

### The role of the α_IIb_ subunit cytoplasmic domain in outside-in signaling

**Figure 5:**
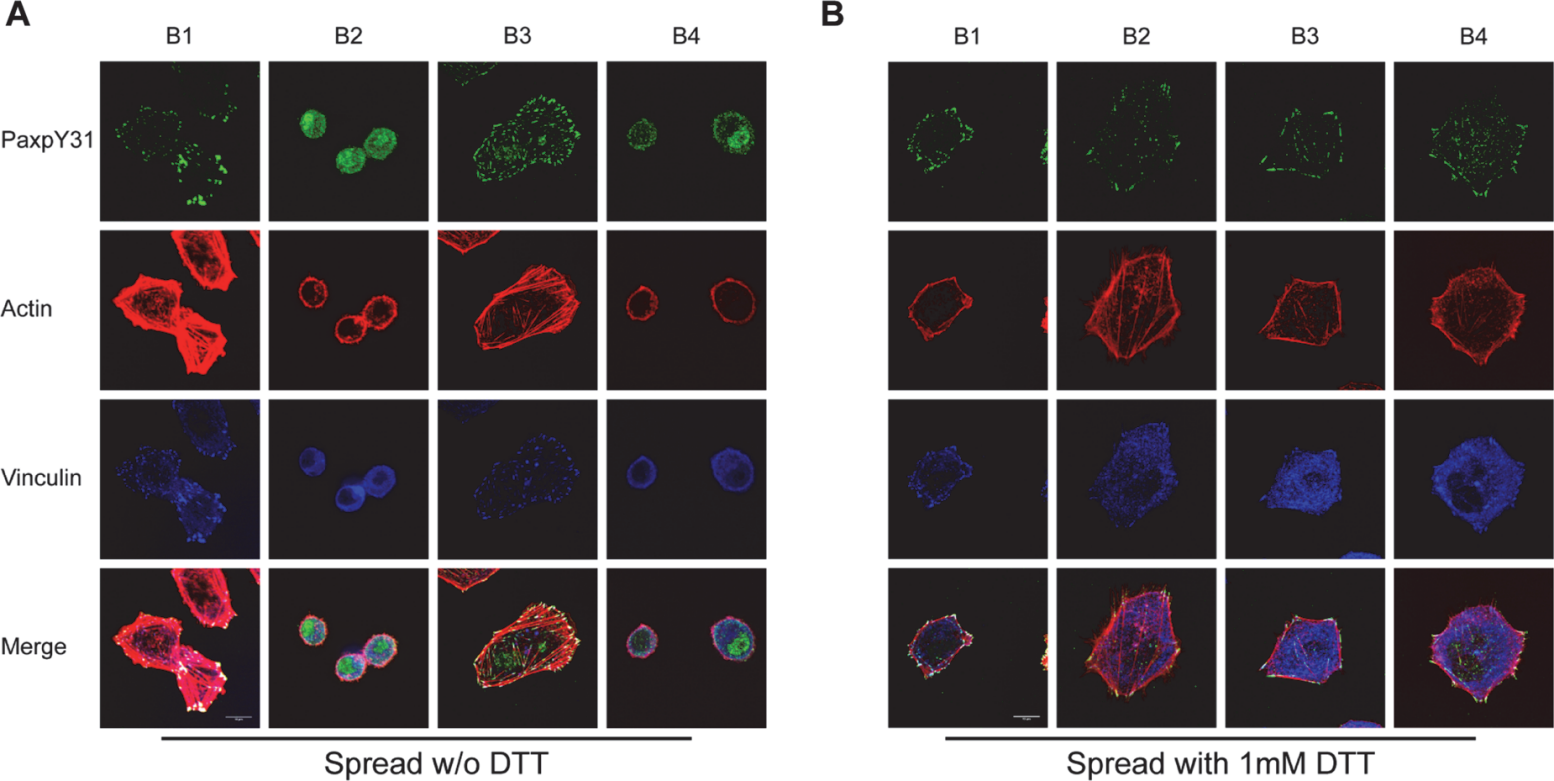
TM domain separation but not α_IIb_ cytoplasmic domain is required for recruitment and phosphorylation of paxillin. (A) Recruitment of phosphorylated paxillin (PaxpY31, green) to FA sites (blue) was observed in both wild type (B1) and α_IIb_-truncated mutant (B3) but not in the two disulfide-bonded mutants (B2 and B4). Note that recruitment of phosphorylated paxillin was normal in the α_IIb_-truncated mutant indicating a dispensable role of the α_IIb_ cytoplasmic domain in paxillin recruitment. (B) Addition of 1mM DTT restored recruitment of phosphorylated paxillin in both of the disulfide-bonded mutants. White bar: 10μm.

We reported earlier that FAK activation and recruitment were normal in both WT and the α truncated mutant (Fig. 4C). Since the recruitment of activated FAK (FAKpY397) to FA sites is considered to be an early signature event of outside-in signaling [30], our observation implied that the α_IIb_ cytoplasmic domain is dispensable in early stages of outside-in signaling. We then asked if the α_IIb_ cytoplasmic domain is involved in a somehow later event, i.e. paxillin signaling. Phosphorylation of paxillin at tyrosines 31 and 118 was reported to be essential for lymphocyte migration [41,42]. If α_IIb_ integrin is involved in paxillin signaling, deleting its cytoplasmic domain would result in both defective paxillin recruitment and reduced paxillin phosphorylation. We found that recruitment of phosphorylated paxillin (pY31) to FAs (Fig. 5A) in both wild type (B1) and the α truncated mutant (B3) were similar, indicating that the α_IIb_ integrin cytoplasmic tail is dispensable for paxillin recruitment and phosphorylation. Because both disulfide-bonded mutants (B2 and B4) exhibited defective phospho-paxillin recruitment, which was restored upon DTT treatment (Fig. 5B), we concluded that TM domain separation is essential for FA signaling transduced through paxillin. We also determined the activation of Extracellular Signal-Regulated Kinase 1 and 2 (Erk1/2) during outside-in signaling because the MAPKs were reported to participate in paxillin-mediated signaling and cell survival [43,44]. We found that Erk1/2 activity was significantly lower in the two disulfide-bonded mutants than in the WT and the α-truncated mutant when adhering to immobilized Fbg (S3 Fig.). This provided further evidence that TM domain separation but not the α_IIb_ subunit is required for paxillin signaling and downstream signaling.

### Kinases and outside-in signaling

**Figure 6:**
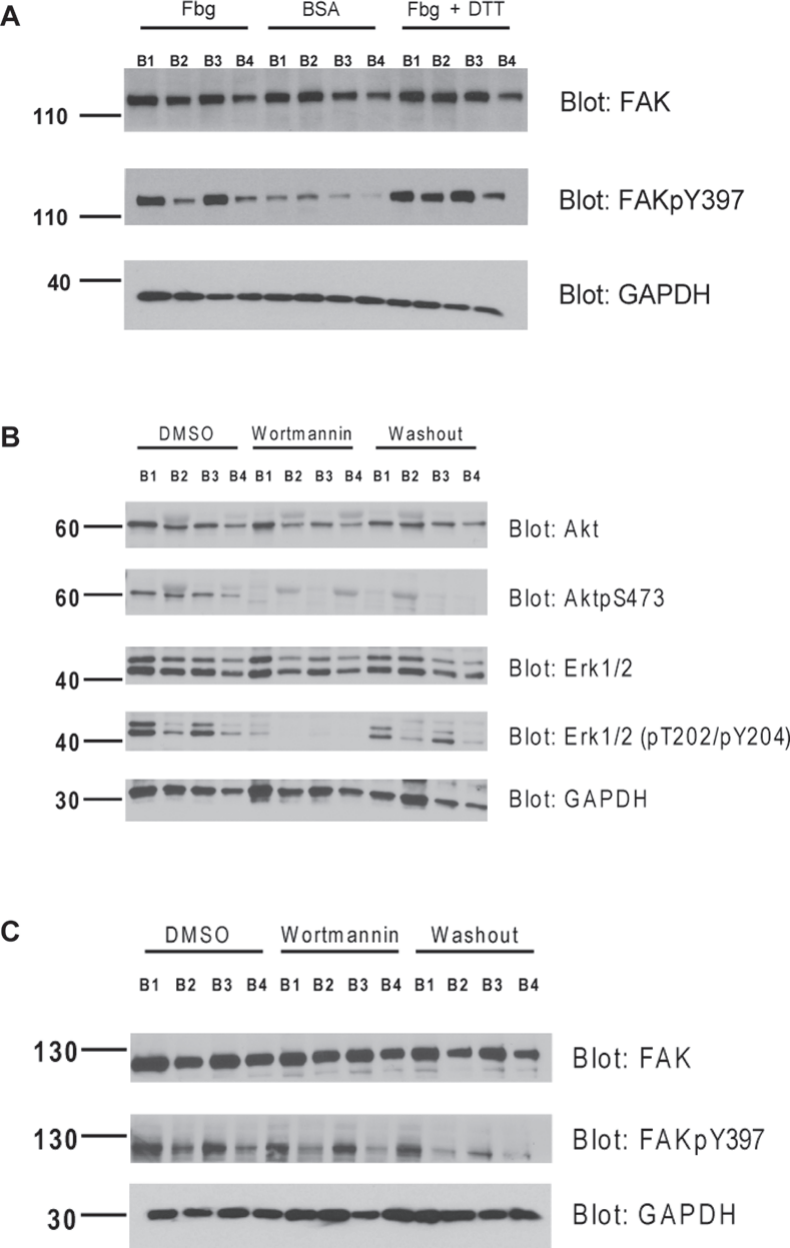
TM separation promotes activation of FAK whereas PI3K regulates Akt and Erk1/2 but not FAK in outside-in signaling. Cells were seeded on Fbg (20μg/mL)-coated dishes with or without 1mM DTT or with 1.5μM/mL Wortmannin at 37°C for 1h and then lysed and subjected to western blot. Cells seeded on 1% BSA coated dishes were used as control. (A)FAK activation induced by immobilized ligand Fbg is TM domain separation dependent. TM-clasped mutants expressed reduced phosphorylation on Y397. (B) Treatment with Wortmannin ablated Akt and Erk1/2 activation. Removing Wortmannin before cell spreading restored Erk1/2 activation without inducing Akt activation. This may implicate that PI3K but not Akt activity is required for cell survival and proliferation mediated by Erk1/2 in outside-in signaling. (C) Wortmannin treatment did not alter activation of FAK, implicating parallel pathways in outside-in signaling.

In our study, FAK activation (phosphorylation on Y397, Fig. 6A) was observed in WT (B1) and α-truncated mutant (B3), but not in the disulfide-bonded mutants (B2 and B4). DTT treatment but not α_IIb_ cytoplasmic domain truncation restored FAK activation in both disulfide-bonded mutants, suggesting that the TM separation rather than α_IIb_ cytoplasmic domain is essential in regulating FAK activity.

**Figure 7:**
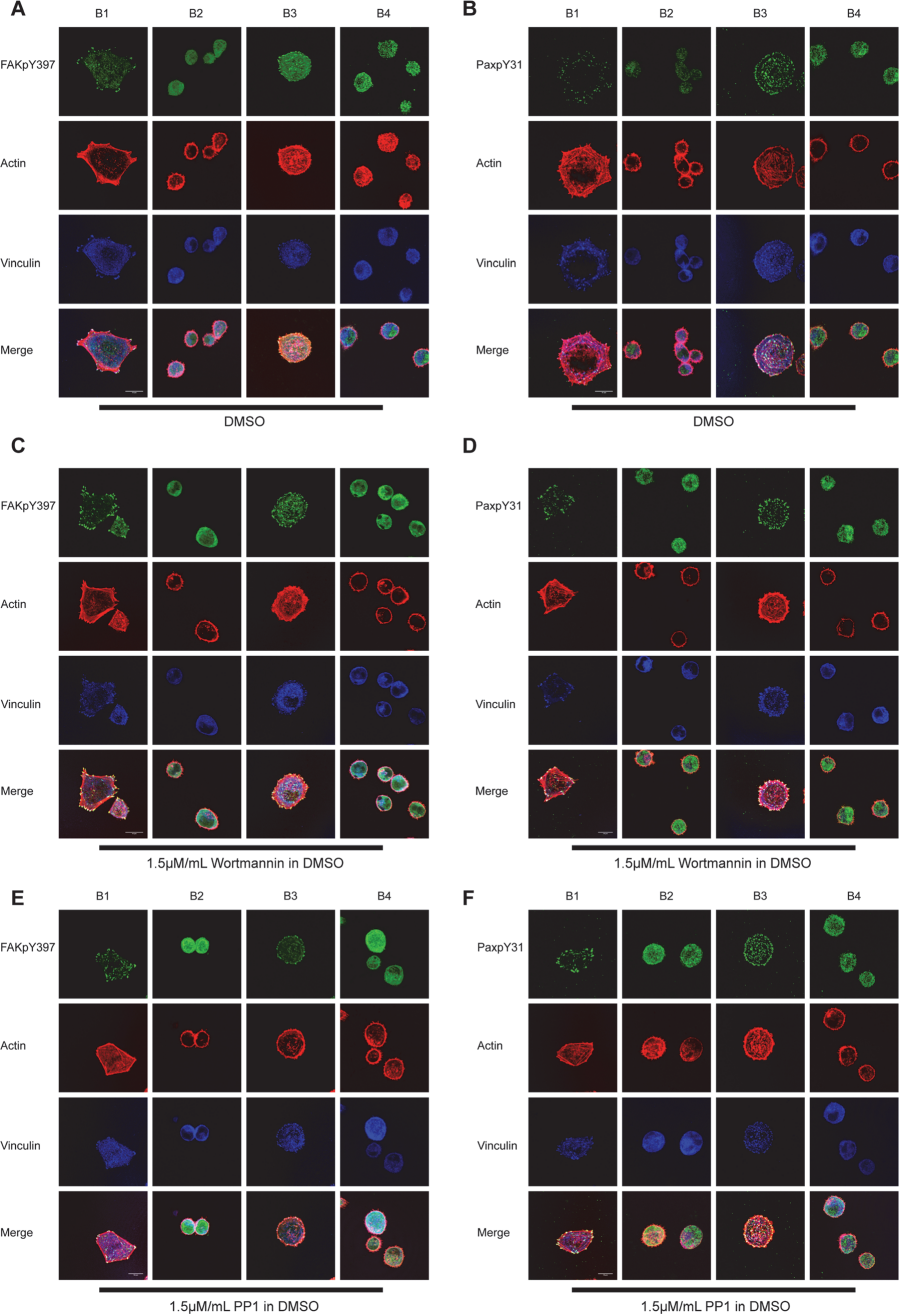
Inhibition of PI3K and Src did not affect cell spreading, FAK/paxillin recruitment, or phosphorylation. (A, B) FAK and paxillin were phosphorylated and recruited to FAs normally in WT (B1) and the α_IIb_-truncated mutant (B3) with control treatment (DMSO). TM clasping abolished the events as previously observed; (C, D) Treatment with 1.5μM/mL Wortmannin did not affect recruitment of phosphorylated FAK and paxillin. (E, F) PP1 (15μM/mL) had little to no effect on cell spreading with regard to FAK/paxillin phosphorylation and recruitment. White bar: 10μm.

The PI3K/Akt pathway has been reported to be involved in integrin-mediated signaling [32,33], and Akt specifically has been reported to be activated during outside-in signaling in platelets [45,46]. However, because this kinase is not directly related to small GTPases, which dictate cell spreading and migration, we hypothesized that the PI3K/Akt pathway might be dispensable for cell spreading but that the pathway may be required for MAPK activation to promote cell survival and proliferation. Indeed, we discovered that treatment with 1.5μM/mL of the PI3K inhibitor Wortmannin had little or no effect on cell spreading (Fig. 7C and D), and both activated FAK and paxillin were recruited to FAs similarly to DMSO-treated cells (Fig. 7A and B). Immunoblot results clearly showed that Akt activation was abolished by Wortmannin treatment (Fig. 6B), whereas FAK activation was not affected by the treatment (Fig. 6C). Erk1/2 activation was suppressed in cells treated with Wortmannin (Fig. 6B). Akt activation was dispensable for Erk1/2 activation because washing out of Wortmannin restored Erk1/2 activation without enhancing Akt phosphorylation at S473 (Fig. 6B). Taken together, the data strongly suggested that the PI3K/Akt pathway is not required for cell spreading and that PI3K but not Akt activity is required for cell survival and proliferation after attachment to the extracellular matrix (ECM).

Activation of Src was reported to be required for Syk activation and essential for Syk-mediated platelet spreading on fibrinogen [47,48]. However, we failed to observe any Src activation and recruitment of activated Src to FAs in any of our cell lines. We also tested whether the Src inhibitor PP1 could influence cell spreading. Cells treated with membrane permeable PP1 (15 μM/mL) for 2 h prior to and during the cell spreading assay showed no observable difference in actin fiber arrangement, FAK/paxillin phosphorylation, or recruitment (Fig. 7E and F). Similarly, treatment with another potent Src inhibitor, PP2 (15μM/mL), also did not affect outside-in signaling (S4 Fig.). Thus, Src activity-dependent outside-in signaling may be tissue specific and more prominent in hematopoietic cells than in other cells.

## Discussion

Our study revealed for the first time that TM domain separation is an essential step downstream of cytoplasmic dissociation in integrin α_IIb_β_3_ transmembrane signaling. Preventing the separation of the TM domain abolished cytoplasmic tail dissociation induced high affinity integrin and immobilized ligand binding triggered outside-in signaling. The discovery strongly suggested that separation of the TM domain couples intracellular signaling cascades with extracellular conformational rearrangement and vice versa. Truncation of the α cytoplasmic tail did not induce high-affinity integrin in a TM-clasped mutant, indicating that dissociation of the TM domain takes place after cytoplasmic domain dissociation. Furthermore, instead of being an intermediate step coupling cytoplasmic domain dissociation, TM separation is absolutely required for outside-in signaling and cannot be bypassed. Our studies showed that the TM-clasped mutant containing a deleted α_IIb_ cytoplasmic tail was defective in outside-in signaling, indicating that TM domain conformational rearrangement is required for assembly of the FA complex on the β cytoplasmic domain. The α_IIb_ cytoplasmic tail, however, does not play a major role in outside-in signaling because the mutant with a cytoplasmic tail truncation could mediate normal outside-in signaling.

The inside-out activation of integrins involves binding of intracellular proteins to integrin cytoplasmic domains, leading to conformational change of integrin extracellular domains. Several models regarding TM rearrangements during integrin inside-out activation have been proposed [49–51]. A variety of studies on the TM domain using FRET [52]; disulfide scanning and computer modeling [19]; mutagenesis [11] and NMR [16,17] revealed that two TM helices associate in the resting state and separate during integrin activation. A more recent study also indicated that talin binding to β_3_ integrin tail can loosen inner membrane clasp and promote TM domain separation [53]. We have proposed that this process may be coupled with the following conformational changes of the integrin TM and cytoplasmic domains: (1) separation of two TM and cytoplasmic helices; (2) following separation, the α subunit helix maintains the similar structure, whereas the β TM domain may undergo a further conformational rearrangement by embedding five to six more residues into the lipid bilayer [15]. This hypothesis is supported by the boundary determination by glycosylation mapping in which monomeric β1 TM domain was used [54]. In addition to the above mechanism, another possible way for integrin activation may come from force transmission from the actin cytoskeleton. It is possible that lateral force exerted on the β cytoplasmic domain by actin polymerization and contraction will pull the β-leg away from the α-leg and further stabilize opened headpiece with the swung-out hybrid domain. The force can cause tilting on the membrane-embedded β TM domain in the plane of the membrane; stabilizing the dissociated helix in the membrane and facilitating force-induced conformational change. We therefore proposed that this β TM domain rearrangement or tilting may be critical for integrin activation and signaling [15]. Mutation and disulfide-scanning studies revealed that TM-clasping can reverse high affinity α_IIb_ GAAKR mutant, an inside-out activated integrin mimic, into resting state [11]. This could be the result of either defective β TM helix rearrangement, i.e. separation and tilting, or spatial hindrance conferred by proximal α_IIb_ cytoplasmic tail which may prevent binding of intracellular activators and building up of cytosol integrin activating stress force. Truncation of the entire α_IIb_ cytoplasmic domain which eliminated any possible cytosol spatial impediment was unable to transmit inside-out activating signals in the TM-clasped integrin mutant. Therefore, our results implicated that β TM helix rearrangement is essential for integrin inside-out activation.

Previous study showed that TM clasping abolishes outside-in signaling [24]. However, since a clasped TM domain likely leads to associated cytoplasmic domain which causes defective FA assembly, it is unclear if TM separation is essential for outside-in signaling or merely an intermediate step that leads to cytoplasmic dissociation. Our study clearly demonstrates that the TM separation itself is an essential conformational change in outside-in signaling. With the entire α_IIb_ cytoplasmic tail removed, it is unlikely there is any spatial hindrance for kinases or other FA scaffold proteins to bind to the β_3_ cytoplasmic tail. Since TM-clasping does not affect integrin clustering [24] and ligands binding affinity, it is most likely that the abolishment of cell spreading and FA assembly is caused by defected TM domain separation and β TM helix tilting instead of impaired α-β cytoplasmic domain dissociation. The separated and tilted β TM helix may be required for placing the β cytoplasmic domain into a position which favors assembly of the FA complex. It is probable that blocking TM separation by a disulfide-clasp can inhibit the tilted rearrangement of the β TM domain. However, further study is requried to prove this hypothesis.

Studies of integrin signaling have largely focused on β integrin, thus the role of the α subunit cytoplasmic domain has remained elusive. It has also been reported that the α integrin cytoplasmic tail participates in outside-in signaling [45,55]. This mechanism, however, may not be required for initiation and transduction of integrin outside-in signaling, but rather could negatively affect cell spreading. In this study, we discovered that the α_IIb_ integrin cytoplasmic tail is dispensable for cell spreading, FAK/paxillin phosphorylation and recruitment, and actin fiber organization, suggesting that β cytoplasmic tail alone is sufficient for transmitting outside-in signaling. However, more evenly distributed and larger (more mature) FAs in cells expressing α-truncated integrin were also observed. The fact indicates that, though α integrin is not required for cell interaction and spreading on ECM ligands, this subunit may be involved in FA turnover and directional cell migration. Our preliminary study suggests defective cell migration in α cytoplasmic domain truncation mutant. However, it is yet to know if this phenomenon is caused by the mutation since a high ligand binding affinity induced by α-truncation may also lead to reduced cell motility. We are planning further studies to better understand these events.

Kinases have long been considered important in integrin signaling. Akt activation in platelets during outside-in signaling has been reported [32,33]. However, there is no evidence that the PI3K/Akt pathway is related to the regulation of small GTPases, which has been shown to be essential for cell adhesion and spreading [29,56]. Therefore, we hypothesized a dispensable role for this pathway in outside-in signaling. Indeed, treatment with the PI3K-specific inhibitor Wortmannin ablated Akt activation but did not affect cell spreading or FAK/paxillin phosphorylation and recruitment; Akt activation was not restored even after removal of Wortmannin. These results strongly suggested that Akt, though may be activated in an integrin-dependent manner, does not play an important role in outside-in signaling. Activation of FAK and Src is thought to be the core part of the pathway [31,57]. However, neither introducing an unactivatable Src mutant [34] nor inhibition of FAK [35] ablated cell spreading. In addition, our study showed that Src inhibitors did not significantly affect FA formation and actin polymerization during outside-in signaling. This evidence implies that activation of the kinases may not be mandatory for firm adhesion and cell spreading. However, the kinases might play essential roles in further downstream signaling such as adhesion-dependent proliferation, directional migration, and tissue development. Further studies are needed to better understand the role of kinases in integrin signaling.

## Supporting Information

S1 Fig More and larger FAs were formed in the α-truncated mutant (B3) compared to the WT (B1). FA counts and area measurement were achieved by ImageJ, as described. Error bars represent FA counts and area measurements from 50 randomly chosen adherent cells.
(TIF)

S2 Fig Both TM separation and α_IIb_ subunit cytoplasmic tail are necessary for cell migration. (A) Cells were seeded on 20μg/mL Fbg- coated 6-well plates and cultured to confluence. Artificial wounds were then created with sterilized tips. Photos were taken every 4 hours, white bar: 50μm. The results represent five independent assays. (B) Normalized quantification of wound closures were done by ImageJ. Error bars represent five independent assays (note that wounds were always completely closed in WT (B1) cells after 12 hrs culture).
(TIF)

S3 Fig Erk1/2 activation in outside-in signaling is TM domain separation dependent. Cells were seeded on Fbg (20μg/mL)-coated dishes and cultured at 37°C for 1 h and then lysed and subjected to western blot. Activation of Erk1/2 was suppressed by TM-clasping (B2) and was not restored by α_IIb_ truncation.
(TIF)

S4 Fig Treatment with Src inhibitor PP2 did not affect cell spreading. Cells were serum starved overnight and treated with PP2 (15μM/mL) for 2 h prior to seeding on fibrinogen pre-coated plates and during the spreading assay. The treatment did not affect FA formation (blue), recruitment, or activation of FAK (A, green) or paxillin (B, green) and actin organization (red). White bar: 10μm.
(TIF)
