# Supplementary figures and images for: Integrin αIIbβ3 Transmembrane Domain Separation Mediates Bi-Directional Signaling across the Plasma Membrane
Source: PLoS One. 2015 Jan 24;10(1):e0116208. doi: 10.1371/journal.pone.0116208 (PMC4305291; doi:10.1371/journal.pone.0116208)

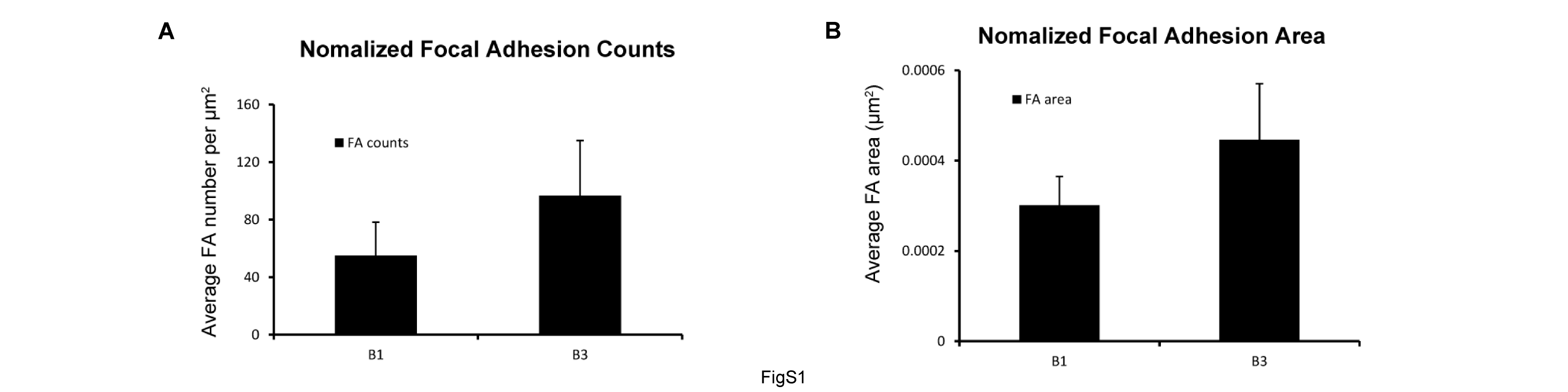

Supplement: S1 Fig — FA counts and area measurement were achieved by ImageJ, as described. Error bars represent FA counts and area measurements from 50 randomly chosen adherent cells. (TIF) [file pone.0116208.s001.tif]

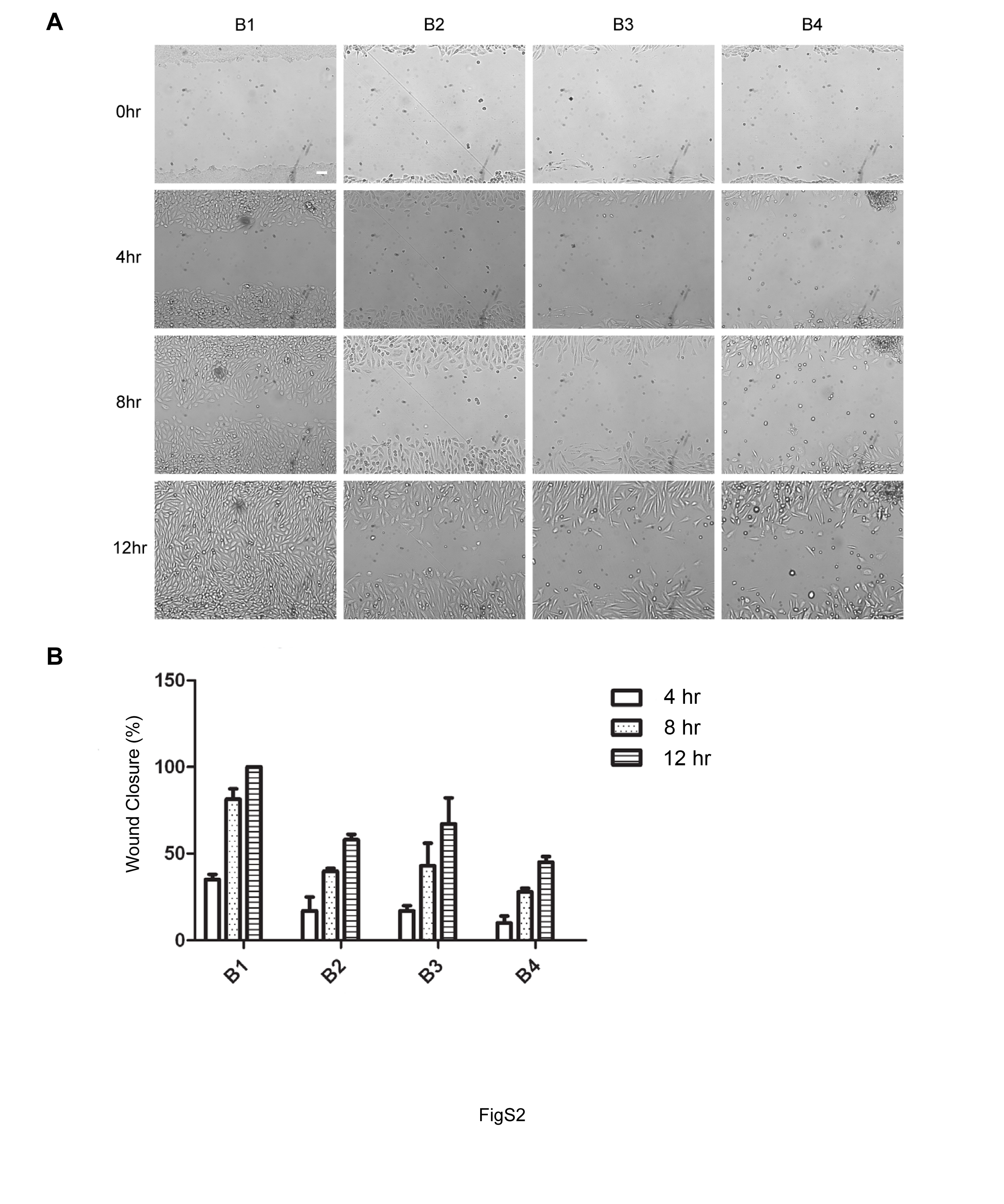

Supplement: S2 Fig — (A) Cells were seeded on 20μg/mL Fbg- coated 6-well plates and cultured to confluence. Artificial wounds were then created with sterilized tips. Photos were taken every 4 hours, white bar: 50μm. The results represent five independent assays. (B) Normalized quantification of wound closures were done by ImageJ. Error bars represent five independent assays (note that wounds were always completely closed in WT (B1) cells after 12 hrs culture). (TIF) [file pone.0116208.s002.tif]

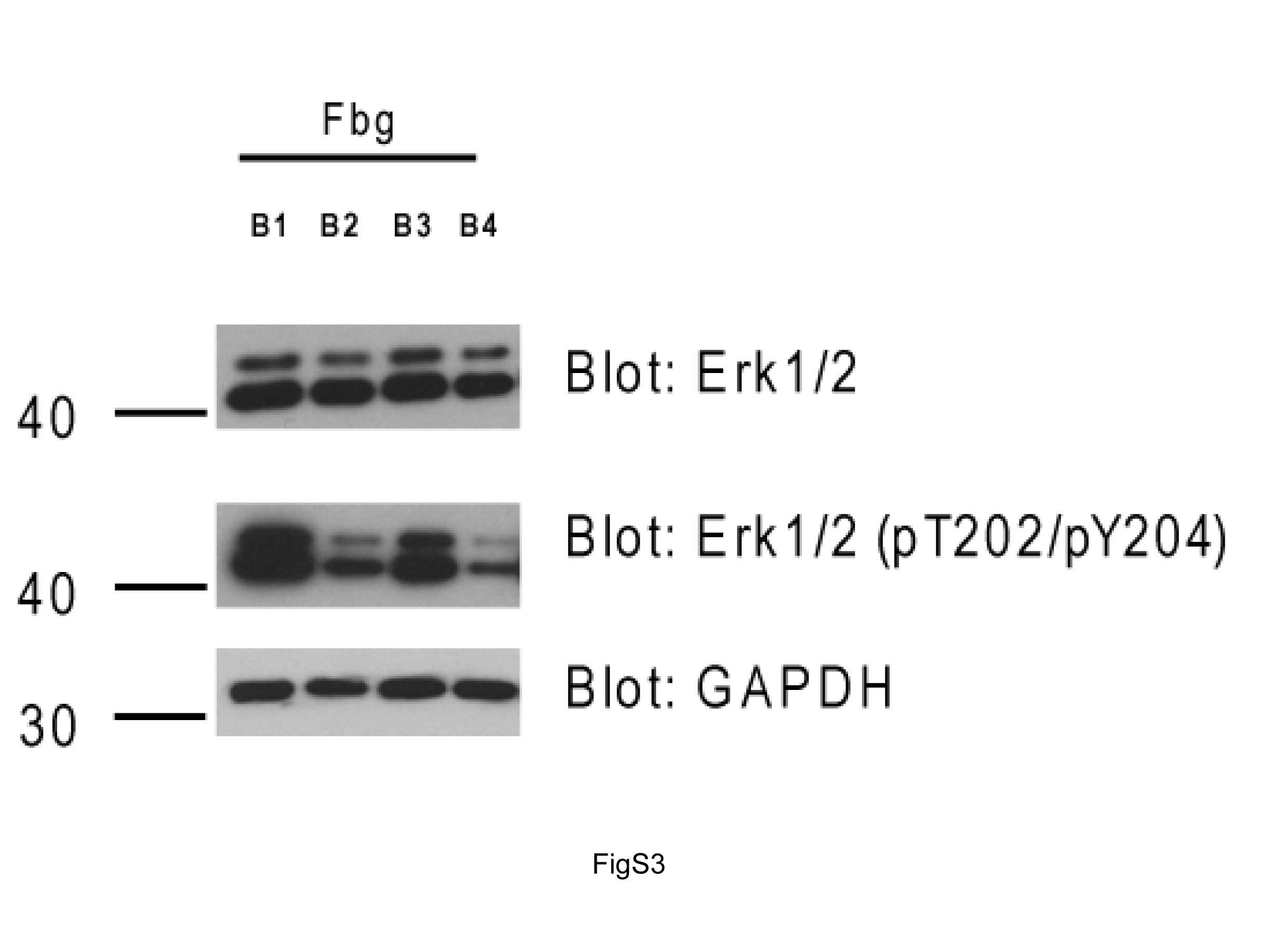

Supplement: S3 Fig — Cells were seeded on Fbg (20μg/mL)-coated dishes and cultured at 37°C for 1 h and then lysed and subjected to western blot. Activation of Erk1/2 was suppressed by TM-clasping (B2) and was not restored by αIIb truncation. (TIF) [file pone.0116208.s003.tif]

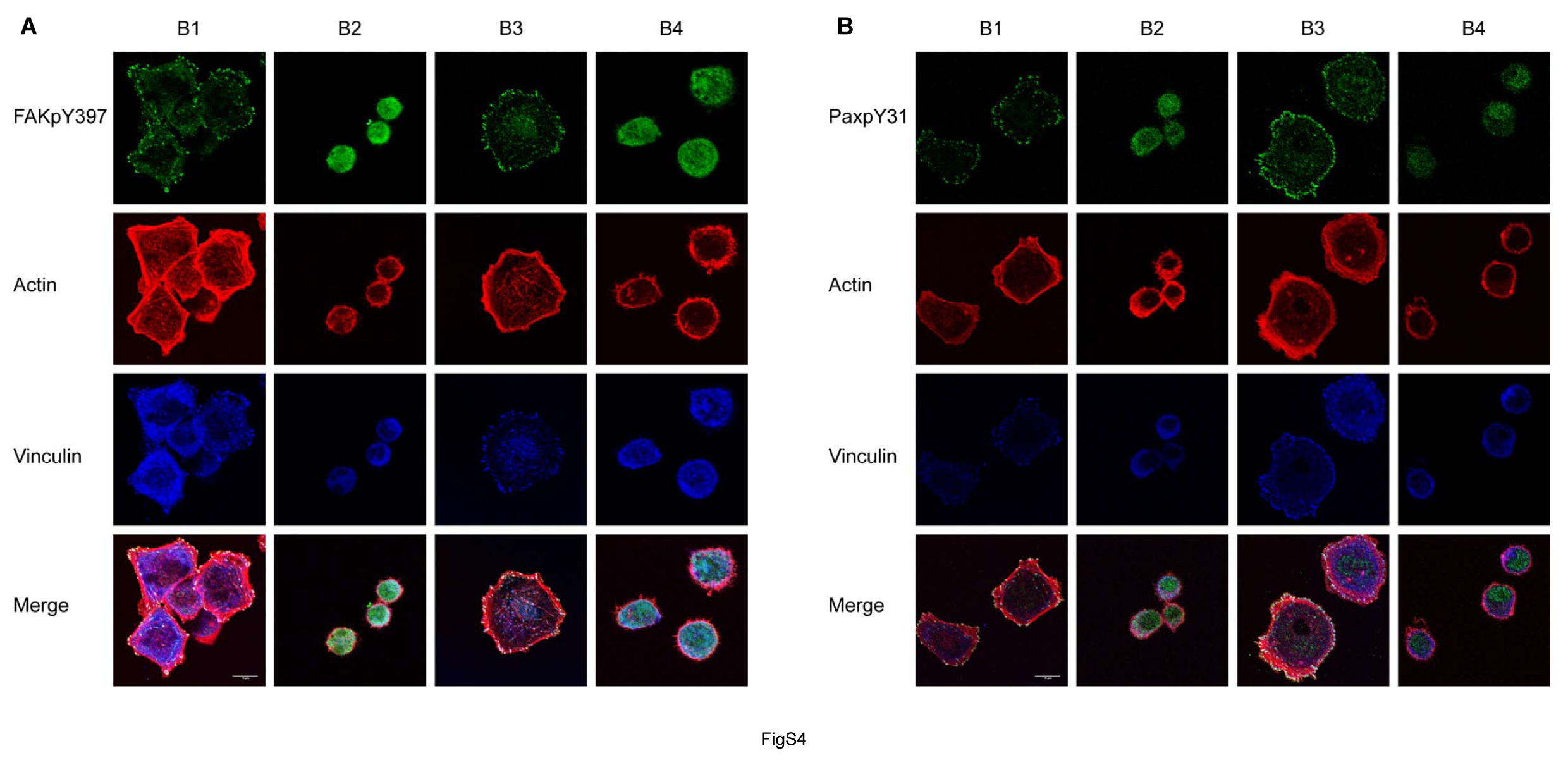

Supplement: S4 Fig — Cells were serum starved overnight and treated with PP2 (15μM/mL) for 2 h prior to seeding on fibrinogen pre-coated plates and during the spreading assay. The treatment did not affect FA formation (blue), recruitment, or activation of FAK (A, green) or paxillin (B, green) and actin organization (red). White bar: 10μm. (TIF) [file pone.0116208.s004.tif]
